# Titanium Alloy Stem as a Cause for Adverse Reaction to Metal Debris after Bipolar Hemiarthroplasty

**DOI:** 10.1155/2014/209461

**Published:** 2014-03-04

**Authors:** Masaaki Sakamoto, Hitoshi Watanabe, Hidetaka Higashi, Hitoshi Kubosawa

**Affiliations:** ^1^Department of Orthopaedic Surgery, Chiba Aoba Municipal Hospital, 1273-2 Aoba-cho, Chuo-ku, Chiba 260-0852, Japan; ^2^Department of Orthopaedic Surgery, Chiba Kaihin Municipal Hospital, 3-31-1 Isobe, Mihama-ku, Chiba 261-0012, Japan; ^3^Department of Pathology, Chiba Aoba Municipal Hospital, 1273-2 Aoba-cho, Chuo-ku, Chiba 260-0852, Japan

## Abstract

A 68-year-old male with failure of bipolar hemiarthroplasty consistent with adverse reaction to metal debris (ARMD) who presented with a painful cystic lesion and lower extremity swelling was encountered. However, revision surgical findings showed no apparent cause of ARMD previously described in the literature, such as corrosion at the head-neck junction and articular abrasion. Therefore, it was difficult to make a definite diagnosis of failure secondary to ARMD, which consequently led to the decision to perform two-stage revision procedure, though the stem was firmly fixed. Postoperative analysis in the retrieval tissues showed that the metal debris mainly originated from the titanium alloy stem itself. Although this is a very rare case, one should be aware that even the well-fixed femoral components themselves have the potential to be the cause of ARMD.

## 1. Introduction

Numerous reports have described failures in hip arthroplasty secondary to adverse reaction to metal debris (ARMD), including osteolysis, metallosis, hypersensitivity reactions, aseptic lymphocyte-dominated vasculitis-associated lesions, cystic lesions, effusions, and pseudotumors [1, 2]. Additionally, there have been various literatures concerning the causes of ARMD. Metallosis has been suggested to occur as a result of either excessive liner wear up to the metal shell, liner breakage, impingement between the femoral neck and the acetabular component, or a combination of these elements [3–5]. Furthermore, numerous reports suggest corrosion at the head-neck junction or neck-stem junction as a cause [6–8]. Concerning the specific causes after hemiarthroplasty (HA), several case reports have been published describing massive metallosis caused by the abraded outer shell or trunnion corrosion in unipolar HA [9–11]. However, in this case, there was no apparent source of metal release previously described in the literature. Quantitative analysis of the metal in the specimens particularly played an important role in identifying the source of metal debris, which gave us new knowledge concerning ARMD.

## 2. Case Presentation

A 68-year-old male with a right femoral neck fracture during bicycle touring underwent bipolar HA (VECTOR-Titan, Peter Brehm, Weisendorf, Germany) elsewhere. The early postoperative course had been excellent, and he was even able to go mountain climbing. Approximately 4 years later, he noticed severe pain especially in the anterior aspect of the right thigh with the onset of swelling in the right lower extremity, and he was referred to our institution for suspicion of infection.

**Figure 1:**
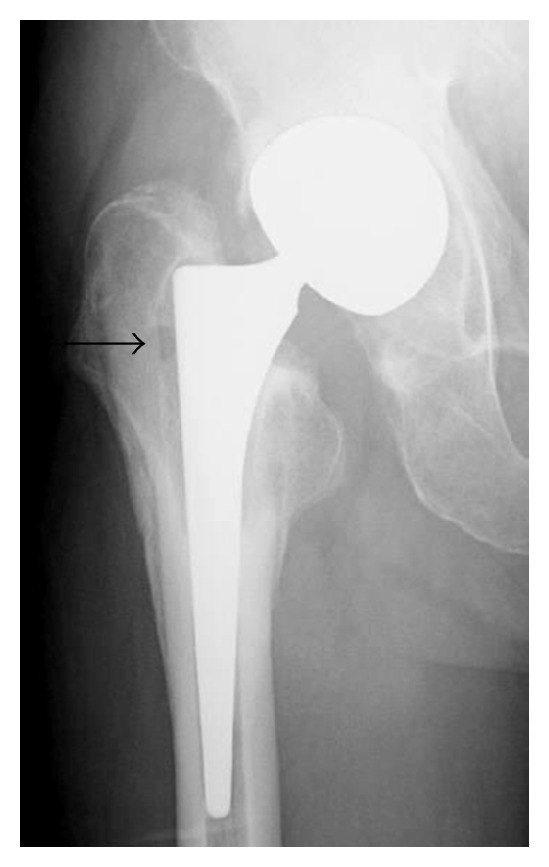
Radiograph showing a circular bone resorption (arrow).

**Figure 2:**
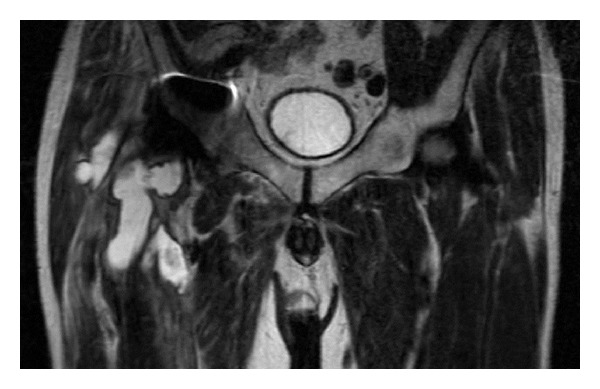
T2-weighted image showing a high intensity lesion with partial low signal intensity, which extends from the lateral aspect of the greater trochanter to the anterior intermuscle of the thigh.

Prior to our consultation, he had no intake of antibiotics. He was unable to walk without the aid of two crutches due to severe pain. Plain radiographs demonstrated evidence of migration of the outer shell, osteolysis, and a circular bone resorption at the proximal part of the femur, but no sinking of the femoral component (Figure 1). Serologic tests showed elevated C-reactive protein at 4.7 mg/dL (0–0.3 mg/dL) and erythrocyte sedimentation rate at 35 mm/hr (0–15 mm/hr), whereas white cell count was normal. Magnetic resonance imaging (MRI) demonstrated a large encapsulated fluid collection (Figure 2). Aspiration fluid from the lesion was reddish-brown in color, but the culture was negative. Serum cobalt and chromium levels were within normal limits, whereas serum titanium level was at the upper limit of 1.0 *μ*g/L.

**Figure 3:**
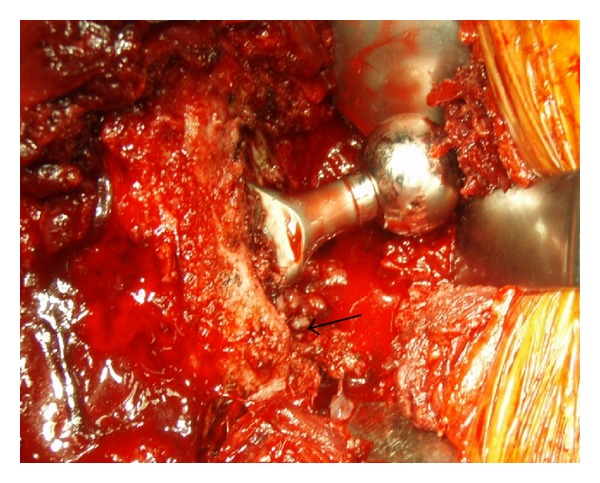
Intraoperative photograph at revision surgery showing granulation tissue at the femoral bone-stem interface (arrow), but no corrosion at the head-neck junction.

**Figure 4:**
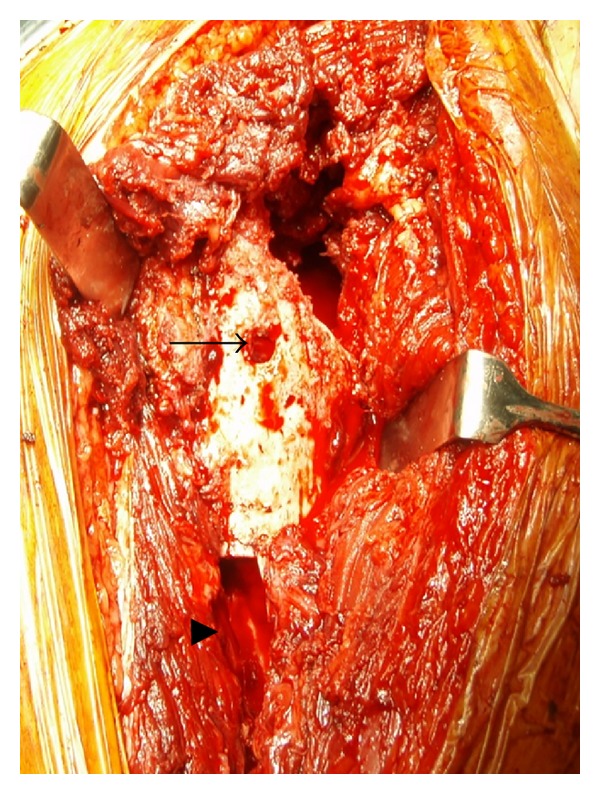
A bone hole at the lateral aspect of the greater trochanter (arrow) and a fenestration of the femur to remove the stem (arrowhead).

At revision surgery, the cystic wall was too thin for retrieval. Macroscopic surgical findings clarified no evidence of apparent metallosis in surrounding tissues, no corrosion at the head-neck junction, no impingement between the femoral neck and outer shell, no massive wear or breakage of the liner, and no outer-shell abrasion. However, granulation tissue was evident at the proximal femoral bone-stem interface (Figure 3). Although the stem seemed to be firmly fixed at least clinically, we decided to retrieve the stem though fenestration of the femur. After the procedure, an erosive bone hole was found at the inner aspect of the femoral marrow cavity, connecting to the lateral aspect of the greater trochanter (Figure 4). Although apparent purulence was not evident anywhere, a one-stage reimplantation was avoided as a precaution to latent infection. Retrieval specimens consisted of tissues from the femoral bone-stem interface, the acetabular bone-cup interface, and the joint capsule, which were submitted separately for cultures and histological examinations.

**Figure 5:**
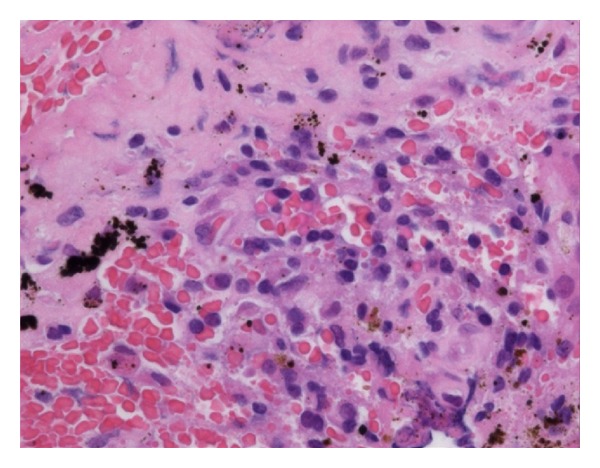
Histological examination showing the metal debris, histiocytes containing metal particles, but no obvious sign of acute inflammation.

All the cultures from specimens were negative for bacteria, and all histological findings were absent of acute inflammation. Further histological analysis revealed existence of metal particles in the specimen from the femoral bone-stem interface (Figure 5), but only a few in other specimens. The amounts of cobalt and titanium in the piece of the femoral specimen were measured by inductively coupled plasma atomic emission spectrometry using SPS5520 (Sumika Chemical Analysis Service, Osaka, Japan). The amount of cobalt was 8 *μ*g, whereas the amount of titanium was 100 *μ*g.

After the improvement of laboratory data and clinical symptoms, reimplantation surgery was performed. During the 1-year followup, no abnormal findings were evident, on repeat radiographs, MRI, and laboratory data.

## 3. Discussion

Revision surgical findings did not coincide with any apparent source of metal release previously described in the literature; it was difficult to make a definite diagnosis of failure secondary to ARMD. However, postoperative analysis of the retrieval tissues excluded low-grade infection. In the specimen from the femoral bone-stem interface, histological findings revealed metal debris and histiocytes containing metal particles, but not polyethylene debris. Hence, it is concluded that metal release led to osteolysis.

Where did the metal debris originate from? Histological analysis confirmed the presence of metal debris in the femoral specimen, whereas metal debris was not obvious in the other specimens. Quantitative analysis of the metal in the femoral specimen showed a much higher ratio of titanium than cobalt. VECTOR-Titan is a cementless stem made of titanium-aluminum-vanadium alloy and has a three-dimensional cone with longitudinal fins to improve the rotational stability. The surface of the stem has a roughness of 40–60 *μ*m. On the other hand, the bipolar cup is made of cobalt-chromium-molybdenum alloy. Therefore, these results indicated that the metal debris mainly originated from the femoral component itself.

Although it was reported that the copious titanium debris could be generated in the case of the unstable component or infection, the question arises as to whether the femoral components themselves in the absence of loosening can release metal debris or not [12, 13]. Jasty et al. [14] described that metal debris may have been liberated locally from the micromovement between the bone and porous coating and discussed that the metal particles may contribute to the production of osteolysis. This supports our case that the femoral component in the absence of loosening has the potential to generate metal wear debris.

To our knowledge, no reports of ARMD associated with the femoral stem itself in the absence of loosening are evident. Presumption through preoperative MRI findings suggests that an erosive bone hole through the greater trochanter may have had continuity with the cystic mass, which was compatible with a symptomatic pseudotumor (Figure 2). However, we could not confirm the continuity during revision surgery, which, unfortunately, is the limitation of this report.

## 4. Conclusion

We believe that osteolysis and a pseudotumor formation in this case were caused by metal release from the titanium alloy stem itself, but not the component junction and the articular surface. One should thus be aware that even the well-fixed femoral components themselves have the potential to be the cause of ARMD. In addition, this fact should be useful in resolving the treatment dilemma of whether to perform one-stage or two-stage revision.
